# Older Adults’ Perceptions and Recommendations Regarding a Falls Prevention Self-Management Plan Template Based on the Health Belief Model: A Mixed-Methods Study

**DOI:** 10.3390/ijerph19041938

**Published:** 2022-02-09

**Authors:** Jennifer L. Vincenzo, Susan K. Patton, Leanne L. Lefler, Pearl A. McElfish, Jeanne Wei, Geoffrey M. Curran

**Affiliations:** 1 Department of Physical Therapy, College of Health Professions, University of Arkansas for Medical Sciences, Fayetteville, AR 72703, USA; 2 Department of Nursing, College of Education and Health Professions, University of Arkansas, Fayetteville, AR 72703, USA; skpatton@uark.edu; 3 College of Nursing, University of Arkansas for Medical Sciences, Little Rock, AR 72205, USA; leflerleanne@uams.edu; 4 Office of Community Health and Research, University of Arkansas for Medical Sciences, Fayetteville, AR 72703, USA; pamcelfish@uams.edu; 5 Department of Geriatrics, Reynolds Institute on Aging, College of Medicine, University of Arkansas for Medical Sciences, Little Rock, AR 72205, USA; weijeanne@uams.edu; 6 Departments of Pharmacy Practice and Psychiatry, University of Arkansas for Medical Sciences, Little Rock, AR 72205, USA; currangeoffreym@uams.edu; 7 Central Arkansas Veterans Healthcare System, North Little Rock, AR 72114, USA

**Keywords:** adherence, patient-centered design, behavioral change, health belief model, patient engagement, shared decision-making

## Abstract

Falls are the leading cause of fatal and non-fatal injuries among older adults. Self-management plans have been used in different contexts to promote healthy behaviors, but older adults’ perceptions of a falls prevention self-management plan template have not been investigated. Using mixed methods, we investigated older adults’ perceptions and recommendations of a falls prevention self-management plan template aligned with the Health Belief Model. Four focus groups (*n* = 27, average age 78 years) were conducted using semi-structured interview guides. Participants also ranked the written plan on paper with respect to each item by the level of importance, where item 1 was the most important, and 10 was the least important. Focus groups were transcribed and analyzed. Descriptive statistics were calculated for item rankings. Older adults felt that the plan would raise awareness and help them to engage in falls prevention behaviors. Participants recommended adding graphics and using red to highlight the risk of falling. Participants opined that ranking the items by level of importance was challenging because they felt all items were important. *‘What might happen to me if I fall’* was ranked as the most important item (average 2.6), while *‘How will I monitor progress’* was the least important (average = 6.6). Considering that older adults need support to engage in falls prevention, future research should investigate the impact of implementing an individually tailored falls prevention self-management plan on older adults’ engagement in falls prevention behaviors and outcomes of falls and injuries.

## 1. Introduction

Falls are the leading cause of emergency department visits, injuries, and hip fractures, resulting in significant morbidity and mortality among older adults over the age of 65 years [1] and costing upwards of $50 billion in direct medical costs annually [2]. Many falls are preventable [3,4], yet approximately 27% of older adults report falling each year [1]. Successful interventions for falls prevention are comprised of single and multifactorial approaches to modify individual risk factors. Evidence-based interventions include, but are not limited to, participation in programs to improve fear of falling, physical function, and balance; management of visual and foot issues, medication management, physical therapy, and home modifications as directed by an occupational therapist [3,4,5]. Implementing even a single intervention could save up to $442 million in direct medical costs [6].

Although falls prevention screening, assessment, and intervention can decrease falls, older adults’ poor adherence to interventions is a significant barrier to addressing the public health issue of falls [7]. Results after community-based falls screenings [8] and a systematic review on fall prevention [9] indicate that at 6 or 12 months, approximately 50% of community-dwelling older adults are likely to adhere to recommended falls prevention interventions. Notably, adherence to health behavior interventions can be improved. For example, supported self-management improves adherence to disease management interventions [10].

Self-management is one of the most frequently used interventions for chronic disease management, especially among people with chronic conditions such as diabetes and heart disease [11]. The concept of self-management refers to the daily management of one’s chronic health conditions [10,11]. Contrary to the implications of the word self-management, it is centered around an individual being an active partner in their healthcare with providers supporting shared decision making, health behavior change, and goal setting. According to the Royal College of Physicians, ‘shared decision-making and support for self-management refer to a set of attitudes, roles, and skills supported by tools and organizational systems, which put patients and caregivers into a full partnership relationship with clinicians in all clinical interactions’ [12]. Shared decision making among older adults and healthcare providers to improve patient engagement in falls prevention has resulted in fewer falls in hospital settings [13,14,15]. However, few studies have investigated shared decision making, patient engagement, or other components of self-management in falls prevention for community-dwelling older adults. Directing efforts of self-management for falls prevention in the community setting is necessary to support an older adult’s choice to age in place [16]. Szanton et al. utilized strategies to engage low-income older adults on chronic disease and physical function self-management in the home-based CAPABLE (Community Aging in Place: Advancing Better Living for Elders) program studies [17,18]. An occupational therapist and nurse worked with the older adult over 5 months using motivational interviewing, shared decision making, goal setting, and individually tailored strategies to assist the older adult in achieving their goals. In one of their many studies, Szanton et al. found that 51% of the older adults chose fall prevention as a goal; of those, 46% fully achieved their goal, and 40% partially achieved this goal [17].

However, studies show that community-dwelling older adults have had challenges with goal setting and achievement. Haas et al. found that Australian older adults who participated in a 15-week home-based or group-based falls prevention program had difficulties setting and achieving goals to facilitate behavior change to prevent falls [19]. Furthermore, healthcare practitioners that supported the older adults had difficulty assisting older adults with goal setting. The STRIDE study (Strategies to Reduce Injuries and Develop Confidence in Elders), the largest pragmatic randomized control trial (RCT) of multifactorial fall prevention in primary care, utilized shared decision making and a fall care plan [20] to promote older adults’ uptake of recommendations but did not follow-up with or support older adults to engage in the fall care plan. As a result, the study results were mixed; older adults’ self-reported injuries from a fall were significantly lower in the intervention group than the control group that received information only, but there were no differences between groups in confirmed fall-related injury [21]. These results highlight the importance of support for self-management, which was also noted in an observational study across four physical therapy practices in New Zealand conducted by Peek et al. [22]. The authors found that physical therapist-supported self-management interventions (e.g., exercise, ice, education) resulted in older adults adhering to recommendations most frequently when the older adults repeated the interventions and when print materials were included with the prescribed interventions. However, there was no patient engagement or shared decision making in the study mentioned above, which may have negatively impacted adherence.

According to the Standards for Reporting Implementation Studies, the background and rationale for a health intervention must be supported by a theory, framework, or model [23]. Implementing health behavior change models to guide interventions can support behavior change and has been successfully used in chronic disease self-management interventions [24,25]. The Health Belief Model (HBM) is a theoretical framework developed to explain engagement in health behaviors using constructs including (1) perceived susceptibility of getting the health condition, (2) perceived severity of the health condition, (3) perceived benefits to taking the recommended action to prevent the health condition, (4) perceived barriers to taking the recommended action to prevent the health condition, (5) cues to action to engage in the health behavior, and (6) self-efficacy to engage in the health behavior [26]. There are several modifying factors in the HBM as well, such as awareness and knowledge regarding the health condition [27]. The HBM is one of the most widely used theoretical frameworks to explain health behavior change [28]. A systematic review found that HBM-based behavior change interventions significantly improve adherence to healthy behaviors [29]. The HBM has also been used in research to predict health behaviors in people with a history of falls [30,31] or design fall prevention interventions [32]. However, no studies have reported using the HBM or another health behavior change framework to specifically support older adults to engage in a falls prevention self-management plan. For example, Kaushal et al. [30] used an expanded model of the HBM, including habits and intention, to identify predictors of physical activity among older adults who have and have not fallen. They found that perceived barriers to physical activity significantly predicted intention to perform physical activity among only people with a history of falls and that cues to action were important predictors of habitual physical activity regardless of fall history. Huang, Tzeng, and Chen [31] used the HBM to predict determinants of engagement in fall prevention activities among community-dwelling older adults. They found that the model predicted 39% of engagement. Of the HBM constructs, self-efficacy had the most significant influence on engagement, which was followed by cues to action, perceived severity, and perceived benefit. Another study utilized the HBM to develop a four-session educational and counseling osteoporosis and falls prevention program for older females. Participants in the HBM-based program demonstrated greater knowledge and adoption of preventive behaviors than a group education-only program that was not based on the HBM [32].

Our research was based on the following: (1) chronic disease self-management plans have been effective in the past, (2) self-management plans need to be refined and tailored to the needs/perceptions of older adults before they can be implemented, and (3) to our knowledge, there is no existing literature regarding the development or implementation of a falls prevention self-management plan, with older adults’ input, based on a behavior change theory. Therefore, we aimed to identify older adults’ perceptions and recommendations of an HBM-based falls prevention self-management plan template. Tailoring a falls prevention self-management plan template for older adults will optimize future studies designed to examine self-management plan implementation.

## 2. Materials and Methods

### 2.1. Design

**Figure 1:**
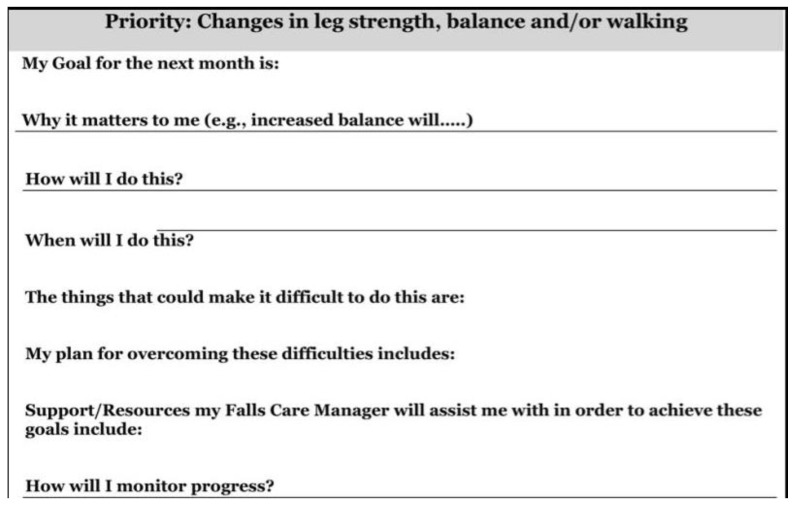
STRIDE study fall care plan for one fall risk item [33].

**Figure 2:**
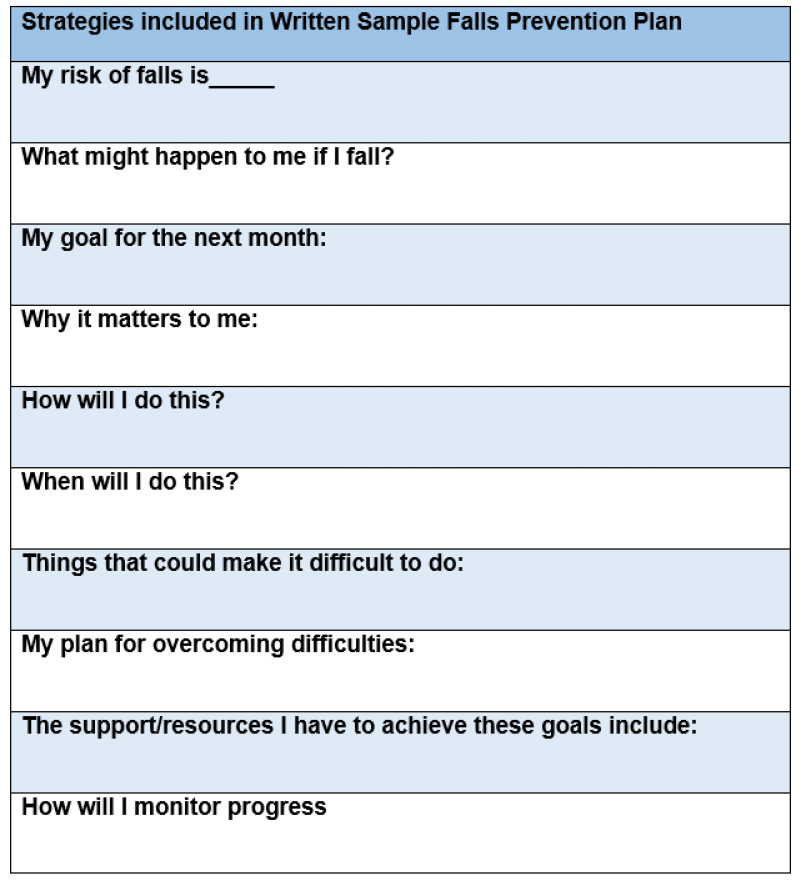
Health-belief model-based falls prevention self-management plan template.

A mixed-methods approach was used to explore older adults’ perceptions regarding a falls prevention self-management plan based on the HBM. The falls prevention self-management plan was adapted from a fall care plan utilized in the STRIDE study, a published pragmatic clinical trial on fall prevention that used stakeholder input, including older adults, to develop study materials, but did not report utilizing a health behavior change theory to inform the plan (Figure 1) [20,33]. For our adaption of the STRIDE fall care plan (Figure 2), we aligned each of the existing 8 items in the STRIDE plan with constructs from the HBM. Then, we added 2 items (my risk of falls is, what might happen to me if I fall) to address missing constructs in the HBM of perceived susceptibility and perceived severity. The plan was in 14-point font for easier reading.

The study team developed and iteratively refined the semi-structured interview guide for the focus groups based on factors related to engaging in health behaviors from constructs in the Health Belief Model (HBM, Table 1; [20,21]). The constructs, perceived susceptibility, perceived severity, perceived benefits, perceived barriers, cues to action, and self-efficacy help explain an individual’s perceptions of the threat of a health problem, the benefits of addressing the threat, and factors that influence the decision to practice a health-promoting behavior. The plan items’ associations with constructs related to the HBM are depicted in Table 1. One-on-one pilot interviews were conducted with three older adults to assess and modify the interview guides. The pilot interview data were not included in the study.

### 2.2. Participant Sampling

Convenience sampling was used to recruit older adults. Individuals who had participated in a prior, unrelated study and indicated an interest in participating in future studies were contacted by telephone. Interested participants were also asked to provide the primary investigator’s contact information to other older adults who might be interested in the research. The inclusion criteria were adults 65 years of age and older, community-dwelling, and ability to attend the focus groups.

### 2.3. Data Collection

Four focus groups of six to eight participants were conducted between December 2019 and February 2020. The primary researcher served as the moderator for three focus groups. A research assistant trained by the primary investigator who attended and documented field notes in the first three focus groups moderated the fourth focus group. Ground rules were established before each focus group. These included being respectful of differing opinions, having only one person to speak at a time, and open sharing of participants’ opinions and experiences [34]. Two focus groups were conducted in a conference room in the primary investigator’s department at the university, one was conducted in a senior center conference room, and one was conducted at the house of a participant per their request. Focus groups were recorded on a digital voice recorder (Olympus WS-853, Olympus America, Inc., Center Valley, PA, USA).

Demographics were collected by written survey. Participants were educated by the interviewer on the falls prevention self-management plan and the purpose of the plan, which was for a healthcare provider to assess the older adult for their fall risk, followed by both the provider and older adult completing the individualized plan together to support the older adult to engage in falls prevention behaviors. Before asking participants about their perceptions of the plan, each participant ranked the 10-item plan on paper with respect to the other items by the level of importance (1–10), where item 1 was the most important, and 10 was the least important. Following rankings, older adults’ perspectives of each item were investigated using the semi-structured interview guide (Table 1, middle column).

At the end of the focus groups, the interviewer summarized general themes regarding the group and participants’ perspectives regarding the appearance and recommendations to improve the plan. Participants were encouraged to provide any feedback or clarification of the summary. The focus groups lasted from 1.25 to 1.5 h. Participants received a $30 gift card for completing the focus group interview.

### 2.4. Data Analysis

Data from the demographic questionnaires and rankings of the falls-prevention self-management plans were entered into Excel, and descriptive statistics were calculated. Audio-taped interviews were uploaded to computerized software (Descript, San Francisco, CA, USA) for initial transcription and edited for accuracy. Data from the focus groups were analyzed to identify, analyze, and interpret meaning based on the focus of the semi-structured interview questions using thematic analysis [35]. Analysis began with a thorough reading and rereading of all transcripts. Two experienced qualitative investigators (JV and SKP) separately read and coded the data. A line-by-line analysis of the data was completed individually to identify important words or phrases regarding the targeted questions, and narratives were applied. Then, investigators met to discuss and agree on the themes, which were perceptions regarding the falls prevention self-management plan and recommendations regarding the falls prevention self-management plan. Perceptions were data and quotes surrounding older adults’ understanding, feelings, attitudes, and interpretation about the plan. Recommendations were data and quotes regarding revisions to the plan. During the final step of the analysis, the investigators reached a consensus on the representative quotes. There were no disagreements among investigators regarding the themes or representative quotes during the analysis phase. Data saturation was obtained when the coders agreed no new information emerged after completing the analysis of the four focus groups [36].

## 3. Results

### 3.1. Sample Description

Table 2 summarizes the participant characteristics. The focus groups consisted of 27 adults with an average age of 78 years and an approximately even distribution of males and females. Participants in the focus groups were engaged and collectively accepting of others’ ideas.

### 3.2. Perceptions Regarding the Falls-Prevention Self-Management Plan

Despite the introduction and description of the plan and how it would be used in practice during the interview, older adults in the focus groups largely did not understand the plan or how it was supposed to be implemented. Participants thought they were supposed to complete the plan based on their current perceived falls risk and what they needed to do to prevent falls while being questioned about the plan. The interviewers reiterated that both the older adult and their healthcare provider would complete the plan after the provider screens them for fall risk. The interviewers’ clarifications assisted the older adults in understanding the purpose of the plan, as evidenced by the feedback older adults provided.

All but one participant liked the plan. Only one participant stated, *“It was confusing to me,”* while others stated, *“I love it,”* and, “*I liked the questions; they’re simple, the terminology is clear to understand.”* Participants felt that all of the items on the plan were *“all important,”* and another stated, *“I saw it as a chronological listing.”*

Participants mentioned how the plan would help with awareness. This was exemplified by statements such as, *“it helps staying aware of it,”* and *“keeping it up … and scheduling it.”* One participant felt that awareness would help with initiating falls prevention behaviors, stating, *“The awareness is the beginning of all action, first [you] have to be aware, and this would help bring awareness to the fact that you need to be sensitive to those possibilities and, and it would get you thinking. And so, it would initiate it, I think.”* Another participant implied that the falls prevention plan increased awareness, *“If you get our attention, you get everybody’s attention. I would think.”*

All participants felt that every item in the plan would help an older adult to implement the plan. Participants felt that no items should be omitted. Participants stated that some of the items might be challenging to answer. This was exemplified by comments such as*, “Why it matters to me. You know? It could be because I want to live a long time and be healthy… maybe a couple of examples might clarify it,”* and, *“Things that would make it difficult- because I’m a caregiver and I don’t have time, or because I’m in pain and it’s so broad that you had to think about it, but you might look at it and say, like, ‘How will I do this?’ You know? How is really big?”*

### 3.3. Discrete Rankings of Items in the Falls Prevention Self-Management Plan

Participants’ discrete rankings of each item on the plan by level of importance (1–10); 1 being the most important item and 10 being the least important are depicted in Table 3. The participants voiced that it was challenging to rank items in the plan by importance because they felt that all items were equally important. Item ranking means ranged from 2.6 to 6.6. Participants ranked the item ‘What might happen to me if I fall?’ as the most important item (ranked 2.6) based on means. Participants ranked ‘*How will I monitor progress*’ as the least important item on the plan (ranked 6.6). The item, ‘*My risk of falls is____’* was most frequently ranked 1, while ‘*How will I monitor progress’* was most frequently ranked 10.

### 3.4. Recommend Revisions to the Plan

Participants suggested a few revisions to the plan. Two participants suggested that examples of items would be helpful, specifically for ‘why it matters to me’ and ‘things that could make it difficult to do.’ Another participant felt it should be a bit shorter, *“I think something maybe not as comprehensive … but more of an abbreviated, maybe three or four questions that the doctor went over with you and your visit, I think would make me more aware of possibilities that may happen. Some accidental fall and then prevent that, and the consequences of it.”* Participants felt that the plan *“needs graphics”* or *“pictures.”* One participant stated emphatically, *“I’m a great believer in graphics.”* Another participant felt graphics would help participants grasp concepts.

*“a lot of times, a personality is something people cannot grasp on their own unless they see something that really points it out. And if you had a graphic showing how many, what the risk of falls were on an age line or something like that, to where they would see that there’s an urgency here, they can place themselves within this data.”*

Participants also had suggestions with regard to colors. One participant felt that *“the separation with the color helps.”* Two others recommended including the color red; *“Well, I think red is always a high alert. So maybe if there was one question that was more important, probably a red color would catch your attention,”* and *“In my case, I would say that number one, risk of falling is red certainly if it’s 10% is still a red.”*

## 4. Discussion

We investigated older adults’ perceptions and recommendations of a falls prevention self-management plan based on constructs from the HBM. This work serves as a first step toward developing a health behavior change-based falls prevention self-management plan template, with stakeholder input, for community-dwelling older adults. Participants liked and had positive feedback regarding the 10-item falls prevention self-management plan. They felt that the plan would raise awareness and help them engage in falls prevention behaviors. Older adults had a few recommendations to improve the appearance of items on the falls prevention plan, suggesting adding graphics and highlighting items such as the risk of falling.

We investigated older adults’ rankings of the plan’s most and least important items. The most important item, on average, was, *‘What might happen to me if I fall,’* which aligns with the HBM construct of perceived severity. The item most frequently ranked at number one was, ‘*My risk of falls is___,’* which aligns with the HBM construct of perceived susceptibility. Studies show that older adults are more likely to engage in falls prevention behaviors if they perceive they are susceptible to falls or injuries (e.g., perceived severity) [37,38]. Huang et al. found that the perceived severity of suffering a fall predicted engagement in falls prevention behaviors, among other constructs [31]. Hill et al. [39] found that self-perceived risk of falls and injury (e.g., HBM constructs of perceived susceptibility and perceived severity) were predictive of engagement in falls prevention. Taken together, these results and supporting data support the inclusion of the items *‘My fall risk is,’* and *‘What might happen to me if I fall*’ in the self-management plan.

Participants ranked the three least important items on the plan as, *‘How will I monitor progress,’* ‘*My goal for the next month is,’ and ‘Things that could make it difficult to do.’ ‘How will I monitor progress’* aligns with the HBM constructs of self-efficacy and cues to action, and *‘My goal for next month is’* aligns with self-efficacy. Despite these being the lowest-ranked items on our plan, Huang, Tzeng, and Chen [31] found that the HBM construct of self-efficacy had the most significant influence on falls prevention engagement followed by cues to action, perceived severity, and perceived benefit. Although we did not measure self-efficacy, the participants’ perceived self-efficacy may have affected their ranking of this item on the plan. Indeed, a meta-analysis found that self-efficacy has a medium effect size (*d* = 0.47) on health-related behavior change [24]. Considering that goal-setting and monitoring progress were the lowest ranked items, and self-efficacy plays a significant role in older adults’ engagement, these results may be due to older adults’ need for support to set and achieve goals and self-management. Indeed, a systematic review of behavior change techniques’ effect on older adults’ self-efficacy and physical activity found that interventions involving unsupported goal-setting and self-monitoring were associated with lower levels of self-efficacy and physical activity [40]. Haas et al. [19] found that Australian older adults had difficulties setting and achieving goals to facilitate behavior change to prevent falls. Conversely, Taylor et al. found that 50% of their CAPABLE participants chose fall prevention as their goal, and approximately the same percentage achieved that goal with the goal-setting support, decision-making support, and strategies interventions in the program [17].

The item, *‘Things that could make it difficult to do’*, which aligns with HBM constructs of perceived barriers, was the 3rd lowest ranked item on the plan. In addition, focus group participants indicated that this item would be hard to address. Kaushal et al. found that perceived barriers to physical activity significantly predicted intention to perform physical activity among people with a history of falls and that cues to action were important predictors of habitual physical activity regardless of fall history [30]. A scoping review on the validity of HBM constructs to predict behavior change, of which only four studies met the inclusion criteria, found that the perceived barriers and benefits were the strongest predictors of behavior change [41]. It is possible that the utilization of shared decision-making and self-management support to identify and overcome barriers may make this item important for the plan. Taken together, these three lowest-ranked items on the plan are still important to include in the plan. Still, research involving the implementation and revelation of the plan will be necessary to provide future directions.

Our research study does have strengths and limitations. Strengths of our study include that it is the first, to our knowledge, to investigate older adults’ perceptions of a falls prevention self-management plan based on the HBM. Limitations to our study are that the sample is homogenous and small; therefore, the generalizability of our results to different cultures and backgrounds may be limited. A small percentage (37%) of our sample had a history of falls, which may have impacted our findings. People with a history of falls may be more fearful of doing activities and restrict themselves in an effort to decrease their risk, which may impact their perceptions of a falls prevention self-management plan and willingness to participate in falls prevention. Future research should compare the differences in falls prevention plan perceptions of older adults with and without a history of falls. It is important to note that the items ranked as the most important on the plan were the first two in order on the written plan. The item ranked as the least important on the plan was the last item in the order, although these items were ranked almost a whole point(s), lower or higher than other items comparatively. In addition, we did not ask participants why they ranked the items in the order that they did. Considering the order of the rankings and that the participants felt that all items on the plan were important and challenging to rank discretely, the rankings should be interpreted with caution.

## 5. Conclusions

Research shows that education alone does not consistently increase engagement in health behaviors among community-dwelling older adults [26] nor does it decrease falls in the home post-hospitalization [31]. Falls prevention in hospital settings is most effective when older adults are engaged in shared decision making [14] and in the community setting when older adults participate in shared decision making and have support for engagement [15,18]. Self-management plans facilitate shared decision making and support for engagement [10]. Our research indicates that a falls prevention self-management plan based on the HBM is viewed positively by older adults. Older adults also feel this plan would help increase their awareness and engagement in falls prevention behaviors. Future studies should investigate the impact of implementing the falls prevention self-management plan on older adults’ engagement in falls prevention behaviors and outcomes of falls and fall-related injuries.

## Figures and Tables

**Table 1:** Interview guide for falls prevention plan guided by constructs of the Health Belief Model.

Items Included in Written Health Belief Model-Informed Falls Prevention Self-Management Plan	Questions: We Are Seeking Your Opinion on the Following: How Does or Does Not This Sample Falls Prevention Plan Help Older Adults:	Constructs of the Health Belief Model
My risk of falls is_____	Understand and acknowledge their falls risk?	Knowledge, perceived susceptibility
What might happen to me if I fall?	Understand what may happen if they suffer a fall?	Perceived threat, perceived severity
My goal for the next month:	Promote engagement and goals?	Self-efficacy, cues to action
Why it matters to me:	See the personal benefit to falls prevention?	Benefit, personal relevance
How will I do this?	Promote engagement and goals?	Perceived facilitators and barriers, self-efficacy
When will I do this?	Make a plan?	Cues to action, likelihood to take action
Things that could make it difficult to do:	Address barriers to undergoing falls prevention?	Perceived barriers
My plan for overcoming difficulties:	Provide facilitators to undergoing falls prevention?	Perceived facilitators, self-efficacy
The support/resources I have to achieve these goals include:	Provide resources and social support to achieve goals?	Cues to action, self-efficacy, social support
How will I monitor progress	Provide accountability to goals?	Cues to action, self-efficacy, social support

**Table 2:** Participant demographics.

Characteristic	*n* (27)
**Sex**	
Male	13
Female	14
**Age ^a^**	
Male	79.4
Female	76.1
**Race/Ethnicity**	
Non-Hispanic—white	21
Did not state	6
**Educational level**	
Less than a high school diploma	2
High school degree or equivalent (GED)	0
Some college, no degree	11
Associate’s degree	2
Bachelor’s degree	5
Master’s degree	3
Professional degree (MD, DDS, DVM)	0
Doctorate (PhD, EdD)	2
No answer	2
**Yearly income**	
Less than $20,000	3
$20,000 to $34,999	6
$35,000 to $49,999	2
$50,000 to $74,999	5
$75,000 to $99,999	7
Over $100,000	3
No answer	1
**Marital Status**	
Married, or in a domestic partnership	19
Widowed	8
**Experienced a fall in the last year**	
Yes	10
No	17
**Number of falls in the last year**	
None	17
1	2
2	2
≥3	3
No answer	3
**Falls resulting in injury**	
Yes	7
No	6
No answer	14

^a^ average age.

**Table 3:** Discrete rankings of items in falls prevention self-management plan.

Falls Prevention Self-Management Plan Item	Ranking Each Item on the Plan with Respect to One Another with a Discrete Number (1–10); 1 Being the Most Important Item and 10 Being the Least (*n* = 27)
My risk of falls is_____	Range = 1–9 Mean = 3.0 ± 2.8 Mode = 1
What might happen to me if I fall?	Range = 1–10 Mean = 2.6 ± 1.0 Mode = 2
My goal for the next month:	Range = 1–10 Mean = 5.7 ± 3.0 Mode = 8
Why it matters to me:	Range = 1–9 Mean = 4.3 ± 2.2 Mode = 3
How will I do this?	Range = 1–10 Mean = 4.6 ± 2.5 Mode = 4
When will I do this?	Range = 2–10 Mean = 5.1 ± 2.3 Mode = 5
Things that could make it difficult to do:	Range = 1–10 Mean = 5.5 ± 3.1 Mode = 9
My plan for overcoming difficulties:	Range = 1–10 Mean = 5.4 ± 2.9 Mode = 5
The support/resources I have to achieve these goals include:	Range = 1–10 Mean = 5.3 ± 3.4 Mode = 3
How will I monitor progress	Range = 1–10 Mean = 6.6 ± 3.8 Mode = 10

## Data Availability

The data are not publicly available due to ethical restrictions.
